# Splenectomy for Splenomegaly

**Published:** 1914-05

**Authors:** 


Splenectomy for Splenomegaly. J. A. Nixon of Bristol reports the removal of simple hypertrophie spleen from a girl aged 14, with recovery but recently, (18 months after operation) suspicion of pulmonary tuberculosis. He reproduces Johnston's tabulation, up to 1908 from *Annals of Surg.*

E. Hey Groves (ibid.) advocates splenectomy early in Banti's disease and reports a successful case.

| Lesion or Disease. | Cases | Recovered | Died |
|---|---|---|---|
| Idiopathic hypertrophy | 74 | 53 | 21 |
| Idiopathic hypertrophy, ectopic spleen | 64 | 50 | 6 |
| Idiopathic hypertrophy, twisted pedicle | 27 | 19 | 8 |
| Malarial hypertrophy | 149 | 111 | 38 |
| Malarial hypertrophy, ectopic spleen | 40 | 39 | 1 |
| Malarial hypertrophy, twisted pedicle | 12 | 10 | 2 |
| Splenic anæmia | 61 | 49 | 12 |
| Cysts, hydatid | 23 | 19 | 4 |
| Cysts, non-parasitic | 19 | 19 | 0 |
| Leukæmia | 49 | 6 | 43 |
| Tuberculosis | 10 | 8 | 2 |
| Sarcoma | 12 | 9 | 3 |
| Abcess | 9 | 8 | 1 |
| Miscellaneous | 13 | 11 | 2 |
| Wounds and injuries | 150 | 99 | 51 |
| Total | 708 | 514 | 194 |
| Per cent | | 72.6 | 27.4 |

